# Oxytocin Modulation in Mindfulness-Based Pain Management for Chronic Pain

**DOI:** 10.3390/life14020253

**Published:** 2024-02-15

**Authors:** Oytun Aygün, Emily Mohr, Colin Duff, Sophie Matthew, Poppy Schoenberg

**Affiliations:** 1 Laboratoire DysCo, Université Paris 8 Vincennes-Saint-Denis, 93526 Saint-Denis, France; oytun.aygun@univ-paris8.fr; 2 Osher Center for Integrative Health, Vanderbilt University Medical Center, Nashville, TN 37203, USA; 3 Breathworks Foundation, Manchester M4 1DZ, UK

**Keywords:** chronic pain, mindfulness, oxytocin, endocrine system, cytokines, stress physiology

## Abstract

In the context of chronic pain management, opioid-based treatments have been heavily relied upon, raising concerns related to addiction and misuse. Non-pharmacological approaches, such as Mindfulness-Based Pain Management, offer alternative strategies. We conducted a mechanistic clinical study to investigate the impact of an 8-week Mindfulness-Based Pain Management intervention on chronic pain, the modulation of inflammatory markers, stress physiology, and oxytocin, and their interplay with clinical pain symptoms and perception, in comparison to a patient wait-list active control. A total of 65 participants, including 50 chronic pain patients and 15 healthy controls, underwent salivary assays to assess endocrine markers, oxytocin, interleukin (IL)-1b, IL-6, IL-8, tumor necrosis factor (TNF)-a, and dehydroepiandrosterone sulphate (DHEA-S). Psychological assessments were also conducted to evaluate aspects of pain perception, mindfulness, mood, and well-being. Findings revealed significant differences between chronic pain patients and healthy controls in various clinical metrics, highlighting the psychological distress experienced by patients. Following Mindfulness-Based Pain Management, oxytocin levels significantly increased in chronic pain patients, that was not observed in the patient wait-list control group. In contrast, cytokine and DHEA-S levels decreased (not to statistically significant margins) supporting anti-inflammatory effects of Mindfulness-Based Pain Management. The fact DHEA-S levels, a marker of stress, did attenuate but not to statistically meaningful levels, suggests that pain reduction was not solely related to stress reduction, and that oxytocin pathways may be more salient than previously considered. Psychological assessments demonstrated substantial improvements in pain perception and mood in the intervention group. These results contribute to the growing body of evidence regarding the effectiveness of mindfulness-based interventions in chronic pain management and underscore oxytocin’s potential role as a therapeutic target.

## 1. Introduction

Chronic pain, defined as the experience of pain on a scale of 3/10 in severity for more than 3-months, presents a significant healthcare challenge, impacting around 20% of European and American populations [1,2,3]. Chronic pain patients show a vulnerability to psychiatric conditions such as depressive and anxiety disorders [4,5,6], even post-traumatic stress disorder [7], along with a reduced quality of life and increased levels of disability [8]. Chronic pain is also linked to higher levels of anxiety and depression symptoms [9,10,11], as well as impairments in decision-making [12], and executive function [13,14], indicating compromised psychological well-being in chronic pain patients. Although chronic pain is a multi-dimensional issue, opioids have remained a common singular treatment strategy, despite the significant associated risks of addiction and misuse [15,16,17].

Non-pharmacological alternatives such as mindfulness have shown far-reaching multi-dimensional effects on several brain, biologic, psychological, and psychosocial outcomes, that has been examined as a promising treatment for chronic pain management. Previous studies have shown that mindfulness effectively alleviates pain in experimental as well as clinical settings [18,19,20,21,22]. While acute pain serves an adaptive purpose, chronic pain is often the result of maladaptive reorganization within pain-related networks, including the anterior cingulate cortex (ACC) and the periaqueductal gray (PAG) [23,24,25,26,27]. Analgesic effects of mindfulness intervention during heat was associated with greater activation of the subgenual ACC, frontal cortex, and right anterior insula [21]. The amygdala, a key component of the limbic system, further modulates pain pathways through interactions with the ACC and PAG [28,29,30,31]. Greater levels of mindfulness have been associated with the down-regulation of pain perception pathways involving the amygdala and the ACC [21,32,33,34,35]. Mindfulness-based breathing intervention was reported to improve amygdala and prefrontal cortex connectivity, associated with less emotional reactivity to negative visual stimuli [36]. In line with amygdala functions [37,38], mindfulness interventions have been shown to stabilize mood and reduce depressive symptoms in chronic pain patients [39,40,41,42,43,44].

There is also evidence to suggest that mindfulness-based interventions can reduce stress levels in chronic pain patients [45,46,47]. Stress-related biomarkers and pro-inflammatory cytokines may additionally be affected in this process [48,49]. In a 3-day mindfulness meditation intervention, not only perceived stress was reduced but mindfulness meditation further mediated reductions in the pro-inflammatory cytokine interleukin, IL-6, at 4-month follow-up [48]. These findings suggest that reduction of stress may be another mechanism of mindfulness that targets pain reduction. 

However, oxytocin, a neuropeptide with central and peripheral routes of release [50,51,52] could be a key player in how mindfulness may be affecting pain processing. Previous studies suggest that oxytocin is negatively associated with depression and anxiety in chronic pain patients [53] and depressive disorder patients [54], where administration of oxytocin has been shown to reduce activation of the amygdala for negative stimuli [55]. Moreover, oxytocin has been shown to not only improve mood but also influence pain perception [56]. Oxytocin may be altering pain experience through different mechanisms. In one scenario, it could affect the pain experience through a psychological mechanism where oxytocin reduces pain sensitivity by improving mood (depression and anxiety). Alternatively, it could have an analgesic effect by reducing stress [56], since oxytocin is associated with lowered stress markers, such as cortisol [57,58,59]. A further mechanism is that oxytocin could be interacting with the pain networks is through the endogenous opioid system. Multiple animal-model studies report that opioid receptor antagonists impair the analgesic effects of oxytocin [56,60,61,62,63], suggesting that oxytocin binds to opioid receptors. Moreover, oxytocin could also up-regulate the release of endogenous opioids in the brain in the PAG [56].

We used the above frameworks to test the potential of Mindfulness-Based Pain Management as a multifaceted approach to address chronic pain, with potential impact upon mood, stress, inflammation, and oxytocin levels. This report addresses two independent questions; first, the exploration of baseline differences between healthy controls and chronic pain patients with regards to targeted psychological and biological markers, that are presently under-researched, in order to highlight the multidimensional effects of chronic pain. Second, a discrete question explores these particular mechanistic facets with regards to mindfulness-based pain management. We delve into the potential mechanisms through which mindfulness may alleviate chronic pain, including mood stabilization, stress reduction, inflammation control, and oxytocin neurohormonal modulation. We initially hypothesized that Mindfulness-Based Pain Management would dampen inflammation and stress biomarkers, while increasing oxytocin levels, and would have an ameliorative effect on chronic pain symptomatology. Additionally, we hypothesized that oxytocin could be linked to pain modulation in pathways of mood stabilization and/or stress reduction. Understanding such mechanisms can offer new insights into treating chronic pain effectively and informing efficient referral strategies.

## 2. Materials and Methods

### 2.1. Design and Sample

**Figure 1:**
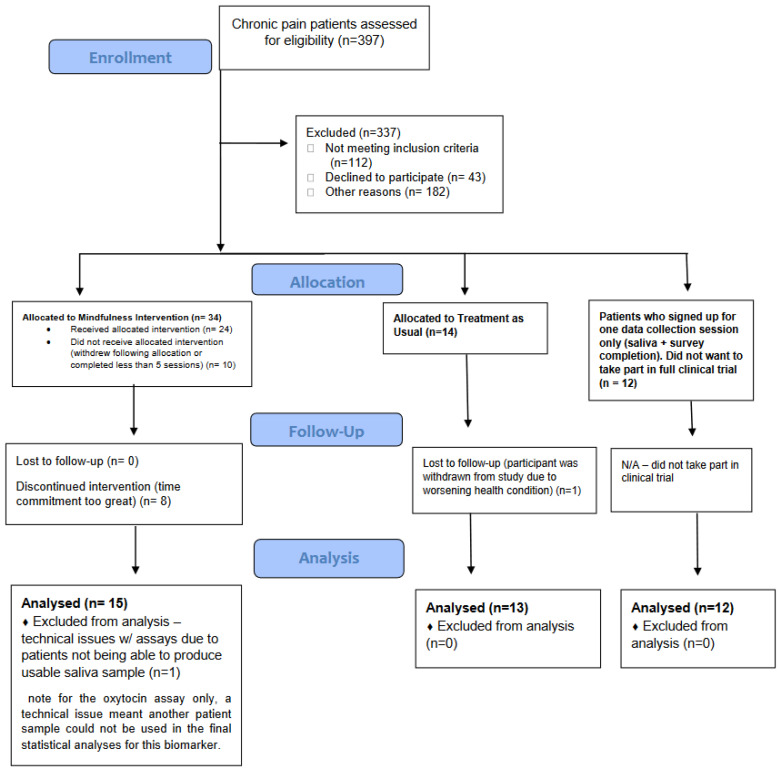
CONSORT schematic of clinical mechanistic trial study implementation.

This mixed-factorial mechanistic clinical study included sixty-five participants; comprising 50 patients with chronic pain, and 15 healthy controls. All groups were matched for age, gender (m/f/non-binary), race and ethnicity. All study procedures were ethically approved by the Vanderbilt Institutional Review Board. The study design was further subsumed into (1) baseline assessments between 50 chronic pain patients (mean age 38.9 years/10.98 s.d.) and 15 matched healthy controls (mean age 33.1 years/10.71 s.d.), and (2) the clinical trial investigation into the effects of mindfulness treatment for chronic pain that included 38 chronic pain patients (of the original 50 chronic pain patients, only 38 signed up for the clinical trial, the remaining 12 provided data during one testing session only to be used for the baseline chronic pain versus healthy control comparisons so to increase sample numbers for analysis since clinical populations can have high variability in biological variables); 24 who underwent the Mindfulness-Based Pain Management intervention, and 14 who were an active “wait-list” control. While it was not possible to conduct true randomization for Mindfulness-Based Pain Management vs. wait-list control group due to logistical reasons, the wait-list control group were chronic pain patients who had no prior experience in meditation/mindfulness but had an interest to attend a mindfulness treatment for their chronic pain in the future. Thus, provided a clinically meaningful patient control group for this study. Due to study drop-outs and issues with providing a quality saliva sample for the assays, the final full dataset usable for statistical analysis included 15 chronic pain patients exposed to Mindfulness-Based Pain Management (mean age 41.3 years/10.61 s.d.) and 13 chronic pain patients in the wait-list control group (mean age 38.6 years/11.25 s.d.), since some patients dropped out from the study and/or did not meet the full attendance requirement for the Mindfulness-Based Pain Management intervention. The 15 chronic pain patients in the Mindfulness-Based Pain Management also lost a further dataset for the oxytocin analyte only due to technical issues with the assaying process. See Figure 1 for a consort schematic outlining the recruitment, follow-up, and final analyses samples.

### 2.2. Inclusion/Exclusion Criteria

Patients meeting the following criteria were invited to participate in the study for the chronic pain group: (a) self-reported pain intensity of 3 on a 10-point numerical scale; and (b) pain present at a similar level for ≥3 months. Exclusion criteria were: (a) patients reporting regular meditation practice (defined as >1 session per week, (b) >10 min per session), (c) left-handedness, (d) history of medical illness associated with possible changes in cerebral tissue (e.g., stroke), (e) neurological abnormalities (e.g., seizure disorder, significant head trauma), (f) Attention/Deficit Hyperactivity-Disorder, and/or (g) psychiatric diagnoses other than anxiety or depression. Since anxiety and depression are often co-morbidities in chronic pain patients, it was first determined if anxiety/depression were primary or secondary symptoms. To be included, patients had to have a primary condition of chronic pain, and any anxiety/depression as secondary symptoms (related to suffering chronic pain).

Healthy controls were excluded if meeting the following criteria: (a) self-reported pain intensity ≥3 on a 10-point scale (b) self-reported regular meditation practice (defined as >1 session per week, (b) >10 min per session), (c) left-handedness, (d) history of medical illness associated with possible changes in cerebral tissue (e.g., stroke), (e) neurological abnormalities (e.g., seizure disorder, significant head trauma), (f) Attention Deficit Hyperactivity Disorder, and/or (g) psychiatric diagnoses.

### 2.3. Mindfulness-Based Pain Management (MBPM) Intervention

Mindfulness-Based Pain Management has been developed by Breathworks^®^ and is an evidence-based group-format standardized treatment program designed for chronic pain. It is a development of Mindfulness-Based Stress Reduction (MBSR), made specifically accessible and sensitive to the needs of a chronic pain population. The overall course structure and format is the same as Mindfulness-Based Stress Reduction but with specific adaptations: (1) mindful activity management and pacing suitable for the chronic pain population where energy and resources can be limited, (2) mindful movements that are gentle and appropriate to the chronic pain population, (3) progressive and sensitive approach to mindfulness of painful sensation, (4) an emphasis on compassion towards a painful body, (5) cultivating a sense of connectedness with others to overcome the isolation that can arise when living with chronic pain. Due to the constraints of COVID-19, sessions were delivered online, where patients met as a group with the instructor once per week via Zoom for 2 h. The intervention included up to 45 min of home practice per day, which was monitored by the instructor as part of the standardized intervention programmatic structure where a portion of group discussion will address the previous week ‘home practices’ and people’s experiences/any questions/challenges/and so on; as well as weekly surveys designed to measure weekly changes in medication regimens and pain levels.

### 2.4. Salivary Data Collection

Proper sample collection was followed according to standardized protocols specified by The Vanderbilt Hormone Assay & Analytical Services Core (VHAC) and Salimetrics Saliva Lab (Carlsbad, CA, USA). Saliva samples were collected using the recommended passive drool method recommended by Salimetrics, and Salivette collection tube (Sarstedt #51.1534.500), that ensured the collection of 400 µL clean saliva to maintain sample integrity and compatibility with almost all analytes. Participants were instructed to not brush their teeth 45-min prior, or have dental work 24-h prior, to sample collection. Samples were stored at −80 °C before being shipped on dry ice to the Salimetrics SalivaLab for analysis.

### 2.5. Endocrine Assay Analysis

The following endocrine markers were analyzed for this study: oxytocin, the following pro-inflammatory cytokines, Interleukins: IL-1b, IL-6, IL-8, and the tumor necrosis factor, TNF-a, as well as the dehydroepiandrosterone sulphate, DHEA-S as a stress marker.

#### 2.5.1. Oxytocin

Samples were assayed in triplicate at the Salimetrics SalivaLab (Carlsbad, CA, USA) using a electrochemilluminesence method developed and validated for saliva by Salimetrics. The average coefficient of variation for all samples tested was <20–30% and exceeds the applicable NIH guidelines for Enhancing Reproducibility through Rigor and Transparency. Sample test volume was 25 μL of saliva per determination. The oxytocin assay had a lower limit of sensitivity of 8 pg/mL, dynamic range from 8 pg/mL–1000 pg/mL.

#### 2.5.2. Cytokines (IL-1b, IL-6, IL-8, TNF-α)

Samples were assayed for the Salimetrics Cytokine Panel (IL-1β, IL-6, TNF-α, and IL-8) in duplicate at the Salimetrics SalivaLab (Carlsbad, CA, USA) using a proprietary electrochemilluminesence method developed and validated for saliva by Salimetrics. The average coefficient of variation for all samples tested was <15%, which meets the SalivaLab’s criteria for accuracy and repeatability in Salivary Bioscience, and exceeds the applicable NIH guidelines for Enhancing Reproducibility through Rigor and Transparency. Sample test volume was 25 μL of saliva per determination. The IL-1β assay had a lower limit of sensitivity of 0.05 pg/mL, with a dynamic range from 0.05–2256 pg/mL. The IL-6 assay had a lower limit of sensitivity of 0.06 pg/mL, with a dynamic range from 0.06–3068 pg/mL. The IL-8 assay had a lower limit of sensitivity of 0.07 pg/mL, with a dynamic range from 0.07–2336 pg/mL. The TNF-α assay had a lower limit of sensitivity of 0.04 pg/mL, with a dynamic range from 0.04–1360 pg/mL.

#### 2.5.3. DHEA-S

Samples were assayed at the Salimetrics’ SalivaLab (Carlsbad, CA, USA) using the Salimetrics Salivary DHEA-S Assay Kit (Cat. No. 1-1252), without modifications to the manufacturers’ protocol. Samples were thawed to room temperature, vortexed, and then centrifuged for 15 min at approximately 3000 rpm (1500× *g*) immediately before performing the assay. Samples were tested for salivary DHEA-S using a high sensitivity enzyme immunoassay (Cat. No. 1-1252). Sample test volume was 100 μL of saliva per determination. The assay had a lower limit of sensitivity of 95.14 pg/mL, a standard curve range from 189–15,300 pg/mL, and an average intra-assay coefficient of variation of 7.25%, and an average inter-assay coefficient of variation 9.51%, which meets the manufacturers’ criteria for accuracy and repeatability in Salivary Bioscience, and exceeds the applicable NIH guidelines for Enhancing Reproducibility through Rigor and Transparency.

### 2.6. Clinical Outcomes

The following self-report surveys were collected from all participants:

#### 2.6.1. Patient Reported Outcomes Measurement Information System (PROMIS-29) v2.0 [64]

A 29-item tool designed to measure self-reported physical, mental, and social health and wellbeing across seven domains: depression, anxiety, physical function, pain interference, pain intensity, fatigue, sleep disturbance, and ability to participate in social roles and activities. Table 1 and Table 2 show the summed PROMIS-29 pain interference subscale scores, where higher scoring indicates higher pain experience/interference (to note, if standardized T scores are used for total PROMIS-29 score, for example, then higher scores represent better health).

#### 2.6.2. McGill Pain Questionnaire—Short Form [65]

Consists of 15 pain-related descriptors (11 sensory, 4 affective and scored on an intensity scale as follows, 0 = “none”, 1 = “mild” and 3 = “severe”) designed to measure a patient’s pain experience. Zero indicates no pain and higher scores indicate the presence of a greater pain experience.

#### 2.6.3. Patient Activation Measure Short Form (PAM-13) [66]

A 13-item scale designed to measure patients’ beliefs, confidence, knowledge, and skills regarding health management. Each item is answered on a 4-point Likert scale, from “strongly disagree” to “strongly agree” or “non-applicable”. Higher PAM-13 scores indicate higher levels of patient activation.

#### 2.6.4. Pain Catastrophizing Scale (PCS) [67]

A 13-item scale designed to measure pain on three subscales: rumination, magnification, and helplessness. Each item is scored using a 5-point Likert scale, from 0 = “not at all”, to 4 = “always”, the scores may range between 0–52, higher scores indicate higher levels of pain behavior and disability.

#### 2.6.5. Beck Depression Inventory (BDI-II) [68]

A 21-item measure that evaluates key affective, cognitive, and neurovegetative symptoms of depression listed in DSM-5. Scores vary between 0–63, higher scores indicate the presence of higher or more severe depressive symptoms.

#### 2.6.6. Multidimensional Assessment of Interoceptive Awareness (MAIA) [69]

A 32-item self-report measure composed of the following 8 subscales: (i) Noticing: awareness of uncomfortable, comfortable and neutral bodily sensations; (ii) Not-Distracting: the tendency to not ignore or distract oneself from sensations of pain or discomfort; (iii) Not-Worrying: the tendency to not react with emotional distress or worry to sensations of pain or discomfort; (iv) Attention Regulation: the ability to sustain and control attention to bodily sensation; (v) Emotional Awareness: the awareness of the connection between bodily sensations and emotional states; (vi) Self-Regulation: the ability to regulate psychological distress by attention to bodily sensations; (vii) Body Listening: actively listening to the body for insight; and (viii) Trusting: experiencing one’s body as safe and trustworthy. Higher scores indicate more awareness of body sensations.

#### 2.6.7. Five Facet Mindfulness Questionnaire (FFMQ) [70]

A 39-item assessment of trait mindfulness encompassing “observing”, “describing”, “acting with awareness”, “non-judging”, and “non-reactivity”. Higher scores indicate the presence of more mindfulness.

### 2.7. Overall Statistical Analysis

Due to the discrepancy in group sizes between chronic pain patients (n = 50) and healthy controls (n = 15) for the baseline comparisons, assumption of homogeneity of variance was tested using the Levene Test. When homogeneity of variance was met, one-way ANOVA was used to compare the baseline means with Group as independent factor. When homogeneity of variance was violated, groups baseline means were compared using the non-parametric Kruskal Wallis H test. For the MBPM trial, each patient provided their own control, due to the pre-post design. A 2× Time (pre, post) × 6× Biomarker (OXT, IL-1b, IL-6, IL-8, TNF-a, DHEA) repeated measures ANOVA tested mean differences between 2× Group (Mindfulness-Based Pain Management group, wait-list control group). Partial Eta squared (np2) effect sizes were also calculated/reported, where 0.01 observes a small, 0.06 medium, and >0.14 large, effect. Post-hoc paired t-tests using Bonferroni correction compared within group differences, with cohen’s *d* calculation for effect size, where >0.2 observes a small, >0.5 a medium, and >0.8 large, effect.

## 3. Results

### 3.1. Baseline Comparison Chronic Pain Patients vs. Healthy Controls

See Table 1 for baseline clinical and endocrine assay data between groups. For the biologic variables/biomarkers, chronic pain patients only yielded significantly higher levels of IL-8: H = 5.976(1), *p* = 0.015, compared to healthy controls.

### 3.2. Pre-Post Mindfulness Intervention in Chronic Pain Patients

See Table 2 for clinical and endocrine measure data for the trial in chronic pain patients exposed to Mindfulness-Based Pain Management versus wait-list control. A significant main effect of Biomarker: F(5,20) = 24.592, *p* < 0.001, np2 = 0.860 was found. Moreover, Biomarker*Group: F(5,20) = 2.910, *p* = 0.039, np2 = 0.421, and Time*Biomarker*Group: F(5,20) = 3.536, *p* = 0.019, np2 = 0.469, interactions were significant. Follow-up paired t-tests revealed that chronic pain patients in the intervention group showed significant increase pre-to-post Mindfulness-Based Pain Management in oxytocin: t(13) = −2.302, *p* = 0.038, *d* = 0.68. There was a contrary was apparent in the waitlist patient control group, where patients showed a reduction tendency (although non-significant) in oxytocin pre-to-post control period. Furthermore, patients exposed to Mindfulness-Based Pain Management generally yielded decreases in inflammatory markers pre-to-post intervention, albeit to non-significant levels, see Table 2 Chronic pain patients in the wait-list control group yielded significant increase in pre-to-post IL-1b: t(12) = −3.084, *p* = 0.009, *d* = 1.01.

## 4. Discussion

In the present study the effects of a Mindfulness-Based Pain Management program on chronic pain were investigated. Potential mechanisms through which Mindfulness-Based Pain Management could be working were explored by measuring stress, inflammation markers, oxytocin levels, as well as psychometric measures of pain experience and mood. The results revealed multiple findings confirming previous studies as well as novel explorations. First, chronic pain patients significantly differed in pretest clinical measures compared to healthy controls. Chronic pain patients scored significantly lower in various health and well-being parameters, while demonstrating higher scores in depression and pain-catastrophizing. Chronic pain patients also had significantly higher cytokine IL-8 levels compared to healthy controls. Psychological assessments after the 8-week mindfulness intervention demonstrated significant improvements in various aspects of pain perception, including decreased pain interference and pain intensity. Additionally, patients exhibited decreased depression symptoms, and enhanced mindfulness traits. Second, oxytocin levels significantly increased in the chronic pain mindfulness intervention group, providing novel evidence that mindfulness-based practices can have an impact on oxytocin regulation. Third, the inflammatory marker IL-1b significantly increased in the wait-list control chronic pain patient group, with a similar trend for IL-6 and TNF-a, while all inflammatory markers measured showed a tendency to decrease in the chronic pain patient group exposed to Mindfulness-Based Pain Management. This pattern of results highlights a possible anti-inflammatory effect of mindfulness in chronic pain. DHEA-S levels, a marker of stress, showed a tendency to reduce in the Mindfulness-Based Pain Management group but did not reach significance, suggesting that the observed pain reduction and improvements in psychological measures in the mindfulness intervention group was not solely related to stress reduction.

Comparison of the chronic pain patient group with healthy controls revealed multiple differences in clinical and psychological measures, highlighting the psychological suffering of patients in line with literature [11,71,72,73,74,75]. Chronic pain patients scored significantly lower in physical, cognitive and social health parameters, further confirming the link of chronic pain to multidimensional health conditions [76,77,78,79]. Pain catastrophizing, which is associated with physical and mental functioning, was found to be significantly higher in chronic pain patients [80,81]. Pain catastrophizing has been defined as an exaggerated negative attitude toward pain experience [67,82] and is linked to negative attitudes toward medical procedures in patients with chronic pain [83,84]. Additionally, chronic pain patients had poorer health management skills and motivation compared to healthy controls, further demonstrating complex difficulties of health management in chronic pain patients, in line with evidence showing impairment in executive functions [13,14] and decision making [12] in chronic pain patients. In our sample, chronic pain patients also had higher scores of depressive symptoms than healthy controls, in accordance with the scientific literature indicating comorbidity between depression and chronic pain [9,11,71,72,74]. Looking at the Multidimensional Assessment of Interoceptive Awareness scale, chronic pain patients scored lower levels in the non-worrying, non-distracting, as well as body-trusting, domains compared to healthy controls. Lower scores of non-worrying and non-distracting could be due to the use of worrying and distracting as methods of coping among chronic pain patients [85,86,87,88]. While lower scores of body-trusting could partially be due to the higher depression seen in chronic pain patients as body-trusting is considered to be impacted in depression [89]. For the biomarkers, chronic pain patients yielded significantly higher cytokine IL-8 levels compared to healthy controls, indicating a potential link between chronic pain and a compromised immune system [90,91].

In the present study the pain experience as well the psychological measures relevant to chronic pain appeared to improve following exposure to the 8-week mindfulness intervention. Self-reported pain experience, measured by the McGill Pain Questionnaire, tapping sensory intensity, cognitive evaluation, and the emotional impact of pain, improved in both groups (i.e., those exposed to Mindfulness-Based Pain Management, and the wait-list control). However, the Mindfulness-Based Pain Management group showed a substantial reduction in pain experience between pre vs. post test measures, while the reductions in pre vs. post test measures of pain for the wait-list control group was less significant, providing further evidence that mindfulness based interventions could be effective in reduction of pain [21,22,41,92,93,94,95]. Previously a short mindfulness meditation-based intervention in healthy participants also showed reductions in pain intensity and unpleasantness in noxious heat induction [21], suggesting mindfulness may enact upon pain pathways. It is also possible that some element of placebo effect may explain the improvement of pain experience for the wait-list control group who also provided weekly check-ins as part of social support. Placebo effects have been shown to reduce chronic pain previously as well [96,97,98]. Although, the higher degree of pain reduction as well as multidimensional changes observed in the other measures observed in the Mindfulness-Based Pain Management group suggest a quantifiable mechanistic effect of the mindfulness intervention. In the present study Mindfulness-Based Pain Management significantly reduced depressive symptoms, that was not present in the wait-list patient control. Chronic pain is associated with anxiety, depression, and other psychological disorders [9,71,72,73,74]. Given that depression is declared among the top causes of disability and death by the World Health Organization [99,100,101], interventions improving depressive symptoms in chronic pain patients is a valuable asset for the biopsychosocial model of health. Other self-measured assessments of health also demonstrated significant improvements in the Mindfulness-Based Pain Management group. Grounded in the World Health Organization’s framework of physical, mental and social health, the patient reported PROMIS-29 showed significant improvements in the Mindfulness-Based Pain Management group, not observed in the wait-list, suggesting a multi-dimensional improvement in overall health and well-being. Furthermore, in the current study the patient’s health management skills and motivation, improved following the mindfulness intervention. A recent study found that mindfulness meditation improves quality of life in chronic pain patients while reducing pain-catastrophizing [102]. However, in the present study we found no changes in pain catastrophizing. Active interventions aiming to develop adaptive psychological attitudes among chronic pain patients, such as exercise, are suggested to reduce the level of pain-catastrophizing [103,104]. It is possible that more physically active forms of contemplative practices such as mindful movement or yoga may be more efficient for pain-catastrophizing [105,106]. Cognitive behavioral therapy could also be an element to consider since pain management interventions based on fear-avoidance models of pain suggest that negative attitudes and beliefs such as pain catastrophizing predict pain-related disability and behavioral avoidance [103,107,108,109,110,111,112]. The Mindfulness-Based Pain Management group also showed higher scores in the Multidimensional Assessment of Interoceptive Awareness/MAIA mindfulness scale; specifically, the Noticing, Attention Regulation, Emotional Awareness, Self-regulation, Body-listening and Body-trusting domains. Previously, it has been shown that mindfulness training is associated with increased MAIA scores not only in patients with depression [113], but also in chronic pain patients with comorbid depression [114], highlighting the psychological benefits of mindfulness training. 

In the present study, perhaps the most important and novel finding is that, after the Mindfulness-Based Pain Management intervention, oxytocin levels significantly increased in chronic pain patients, linking mindfulness to oxytocin regulation. There are only a few studies investigating the role of oxytocin in mindfulness-related practices [115,116], and to our knowledge none investigating the role of oxytocin in the context of mindfulness-based chronic pain management intervention. Previously, it was suggested that oxytocin may impact pain perception through the central nervous system, particularly the limbic system [117], stabilizing mood and reducing depressive symptoms [118]. Here, we found that after the 8-week mindfulness-based pain intervention, oxytocin levels significantly increased while depression symptoms reduced, further suggesting that oxytocin could affect the pain experience through a psychological mechanism where oxytocin dampens pain sensitivity by improving mood [53,54,56].

Furthermore, evidence suggests that oxytocin could affect the pain experience by not only improving mood but also by reducing stress [56,119,120]. The reduction of stress markers observed in other meditation intervention studies [121,122,123] could potentially involve oxytocin, since oxytocin is also associated with lower stress levels measured using cortisol during exercise [57], conflict [58], and/or social stress [59]. Previously Hoehne et al. (2022) investigated the role of oxytocin in stress reduction effects of a socio-affective and compassion-based mental training [116], and found that; although oxytocin levels were affected by the intervention, it did not mediate its stress-buffering effect. In the current study, stress physiology measured by DHEA-S, showed a tendency to decrease but remained non-significant. However, oxytocin increased, suggesting that pain reduction observed in our chronic pain patient sample was not solely related to stress reduction, and that oxytocin-linked analgesia is not only through stress reduction pathways. 

Here, we also found anti-inflammatory effects of the Mindfulness-Based Pain Management intervention, since all the inflammatory markers investigated (IL-1b, IL-6, IL-8, TNF-a), showed decreasing trend, while not reaching statistical significance. This pattern was not observed in the patient wait-list control group, that showed an increase in IL1-b, and trend increases in IL-6 and TNF-a. Taken together, the findings suggest that the Mindfulness-Based Pain Management intervention potentially counterbalanced the negative effects of chronic pain on neuro-immunity, highlighting the compelling anti-inflammatory effects of mindfulness. There is some previous evidence supporting the notion that meditation induces immune response, notably affecting the IL-6 marker, and that this is suggested to be linked to stress reduction [124,125]. In sum, the present findings support a neuro-immune mechanistic effect of mindfulness, at least in chronic pain patients, although further studies are needed to explore whether it is through stress reduction. 

Although the present study added scientific evidence on the effects of mindfulness based treatment in chronic pain and contributed to novel findings, it also had certain limits. This study is among the first to bring evidence that a mindfulness based intervention can increase oxytocin levels. Moreover, this oxytocin increase was associated with reduced pain severity, pain perception, and depression in chronic pain patients. However, we cannot make any assumptions with regards to causality. To do so, further studies are needed to explore the mechanistic role of oxytocin in chronic pain modulation related to mindfulness. Additionally, stress markers showed a tendency to decrease, although they did not reach statistical significance. Thus, further examination of the role of stress reduction upon pain perception associated with mindfulness intervention is warranted. Methodologically, our complete sample had a discrepancy in the number of chronic pain patients versus healthy controls, which could have biased statistical findings in the healthy versus chronic pain comparisons. Furthermore, despite the strength of our longitudinal design, our mindfulness versus wait-list comparisons could have benefitted from a larger sample size overall, since studies with a greater number of participants are needed to confirm our findings and explore longer term effects of Mindfulness-Based Pain Management. The current study presents preliminary data on the effects of oxytocin, potentially through stabilizing mood and linking it to stress pathways. However, it does not explore the potential mechanistic link between oxytocin and the endogenous opioid system. Further studies are needed to expand our understanding of oxytocin in the management of chronic pain with regards to this potential frontier.

Despite its prevalence, chronic pain management has heavily relied on opioid-based treatments, raising concerns about addiction and misuse. Non-pharmacological interventions, such as Mindfulness-Based Pain Management/MBPM, provide alternate potential strategies for treating chronic pain. The present study investigated the impact of an 8-week Mindfulness-Based Pain Management intervention on inflammatory markers, stress physiology, and oxytocin, as well as the clinical pain symptomatology and perception of pain in chronic pain patients. The study puts forward preliminary data suggesting that oxytocin may play a larger role in modulating pain experience and pain perception through mindfulness than previously considered, and highlights its potential as a therapeutic target moving forward. Moreover, the evidence presented suggests that maybe stress is not the primary mechanism of pain reduction through mindfulness. It also presents preliminary data suggesting that mindfulness acts on the inflammatory response. The present study additionally puts forward non-negligible evidence regarding the beneficial psychological effects of mindfulness in chronic pain patients. Future studies are needed to explore the mechanistic intricacies of mindfulness and the potential causality between oxytocin, stress reduction, pain symptomatology, inflammatory response, and psychological effects in chronic pain patients. 

## Figures and Tables

**Table 1:** Baseline Outcome Measures: Chronic Pain Patients vs. Healthy Controls.

	Chronic Pain Patients (n = 50)	Healthy Controls (n = 15)	Statistic *p*-Value (Comparison between Groups)
PROMIS-29	12.50 (3.01)	4.60 (1.24)	<0.001 ***
McGill Pain	16.86 (7.22)	0.43 (1.16)	<0.001 ***
PAM	32.72 (17.24)	3.41 (0.42)	<0.001 ***
PCS	20.44 (9.07)	5.73 (6.80)	<0.001 ***
BDI	13.70 (7.24)	2.57 (2.21)	<0.001 ***
MAIA			
Noticing	3.22 (0.94)	2.77 (1.05)	0.119
Not-Distracting	1.75 (0.97)	2.36 (0.83)	0.032 *
Not-Worrying	2.77 (1.06)	3.51 (0.93)	0.018 *
Attention Regulation	2.56 (0.99)	2.50 (1.13)	0.863
Emotional Awareness	3.13 (1.00)	2.85 (1.18)	0.376
Self-Regulation	2.11 (1.01)	2.58 (1.01)	0.107
Body Listening	1.73 (1.00)	1.56 (0.94)	0.435
Trusting	2.13 (1.22)	3.89 (0.89)	<0.001 ***
FFMQ			
Observing	29.10 (4.28)	23.33 (5.77)	<0.001 ***
Describing	28.48 (5.20)	28.47 (7.86)	0.994
Acting with Awareness	16.86 (7.22)	29.73 (6.50)	0.014 *
Non-Judging	26.67 (5.79)	32.60 (6.48)	0.001 **
Non-Reactivity	20.38 (5.13)	24.40 (5.57)	0.011 *
Endocrine Marker			
Oxytocin	19.80 (21.48)	21.06 (12.92)	0.830
IL-1β	506.22 (831.62)	182.23 (124.09)	0.513
IL-6	8.10 (11.25)	5.45 (5.58)	0.384
IL-8	1697.76 (1634.14)	797.57 (342.53)	0.015 *
TNF-α	9.92 (20.44)	4.62 (3.63)	0.324
DHEA-S	5830.22 (7270.43)	5159.52 (2907.88)	0.729

**Table Key:** PAM, Patient Activation Measure; PCS, Pain Catastrophizing Scale; BDI, Beck Depression Inventory; MAIA, Multidimensional Assessment of Interoceptive Awareness; FFMQ, Five Facet Mindfulness Questionnaire. * *p* < 0.05; ** *p* < 0.01; *** *p* < 0.001.

**Table 2:** Clinical and Endocrine Outcomes in Chronic Pain Patients exposed to Mindfulness-Based Pain Management vs. Wait-list Patient Control.

	Mindfulness-Based Pain Management			Wait-List Control			
	Pre	Post	*p*-Value within Group Comparison	Pre	Post	*p*-Value within Group Comparison	*p*-Value between Group Comparison
PROMIS-29	12.44	9.06	<0.001 ***	11.15	10.69	0.636	0.268
McGill Pain	17.50	9.31	<0.001 ***	13.23	9.38	0.012 *	0.048 *
PAM	42.36	44.66	0.033 *	40.92	41.92	0.875	0.457
PCS	20.56	17.25	0.129	17.00	16.15	0.798	0.296
BDI	13.38	8.56	0.030 *	16.31	14.77	0.475	0.315
MAIA							
Noticing	2.88	3.44	0.027 *	3.56	3.23	0.123	0.060
Not-Distracting	1.35	1.79	0.080	2.03	2.15	0.682	0.069
Not-Worrying	2.90	2.88	0.931	2.85	3.00	0.595	0.898
Attention Regulation	2.39	2.88	0.041 *	2.70	2.58	0.670	0.369
Emotional Awareness	3.19	3.95	<0.001 ***	2.86	2.74	0.565	0.402
Self-Regulation	1.81	3.20	<0.001 ***	2.52	1.98	0.023 *	0.071
Body Listening	1.79	2.88	0.001 ***	1.77	1.97	0.618	0.953
Trusting	1.88	3.04	<0.001 ***	2.59	2.67	0.811	0.120
FFMQ							
Observing	28.81	30.31	0.083	29.46	28.54	0.418	0.699
Describing	27.31	29.56	0.005 **	29.54	30.00	0.746	0.199
Acting with Awareness	22.25	24.63	0.133	26.46	26.31	0.878	0.049 *
Non-Judging	25.38	28.50	0.03 *	27.23	26.15	0.528	0.352
Non-Reactivity	19.81	22.06	0.055	20.62	21.23	0.476	0.698
Endocrine Marker							
Oxytocin	8.81	18.21	0.038 *	20.75	16.63	0.227	0.017 *
IL-1b	902.53	773.43	0.711	116.41	277.58	0.009 **	0.013 *
IL-6	12.37	9.04	0.561	4.60	7.07	0.156	0.163
IL-8	2360.97	2006.30	0.590	1184.93	856.00	0.316	0.097
TNF-a	19.88	6.71	0.153	3.78	6.22	0.081	0.110
DHEA-S	8251.32	4377.27	0.260	3976.77	4559.00	0.519	0.235

**Table Key:** PAM, Patient Activation Measure; PCS, Pain Catastrophizing Scale; BDI, Beck Depression Inventory; MAIA, Multidimensional Assessment of Interoceptive Awareness; FFMQ, Five Facet Mindfulness Questionnaire. * *p* < 0.05; ** *p* < 0.01; *** *p* < 0.001.

## Data Availability

Data will be provided upon reasonable request.
